# Endocyclophotocoagulation

**DOI:** 10.4103/0974-9233.56225

**Published:** 2009

**Authors:** Suzanne M. Falkenberry, Carla J. Siegfried

**Affiliations:** From the Department of Ophthalmology and Visual Sciences, Washington University School of Medicine, St. Louis, Missouri

**Keywords:** Glaucoma, Endocyclophotocoagulation, Cyclodestructive procedure

## Abstract

Endocyclophotocoagulation is becoming a widely accepted and popular treatment of refractory glaucoma and as an adjunct to cataract surgery in both medically controlled and uncontrolled glaucoma. We present a brief review of the indications, technique, safety and efficacy of endoscopic photococylocoagulation.

## INTRODUCTION

Cyclophotocoagulation lowers intraocular pressure by ablating the ciliary processes; thereby, lowering the production of aqueous humor and subsequently the intraocular pressure (IOP). Traditionally, cycloablation was performed transclerally and was reserved for refractory or end stage glaucomas because direct visualization of the targeted ciliary processes was not possible. Side effects include pain, inflammation, chronic hypotony, macular edema, vitreous hemorrhage and phthisis.^1^,^2^ Recently, endoscopic cyclophotocoagulation (ECP) using a diode laser equipped with an endoscope has been introduced. Direct visualization of the ciliary processes permits a more targeted approach.

Histologic studies confirm that there is less tissue disruption associated with ECP than transcleral cyclophotocoagulation.^3^,^4^ A study of rabbit eyes showed that both transcleral and ECP are associated with occlusive vasculopathy, but the endoscopic route is associated with late reperfusion and therefore less chronic poor perfusion.^5^

## INDICATIONS

The use of endocyclophotocoagulation is becoming more accepted and is no longer reserved for end-stage cases.^6^–^9^ Although it is still used for refractory and pediatric glaucomas, ECP is most commonly performed in conjunction with phacoemulsification with intraocular lens placement. Other indications include failure of transcleral cyclophotocoagulation, corneal disorders, aphakic and pseudophakic pediatric glaucoma, history of scleral disease, and plateau iris syndrome.

## DESCRIPTION OF DEVICE

**Figure 1:**
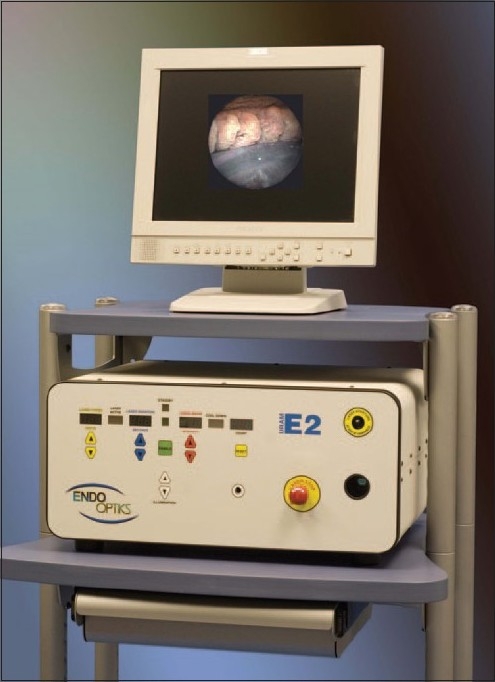
Endoscopic photocoagulation unit. Endo optiks, Little silver, NJ

**Figure 2:**
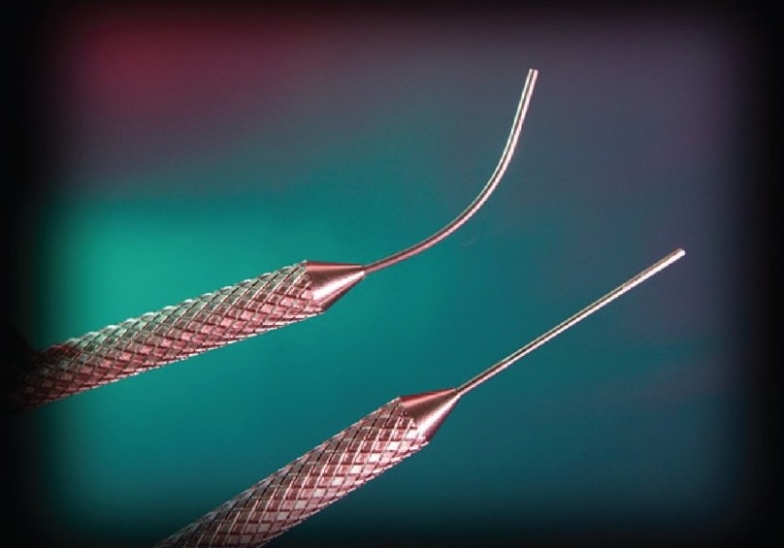
Curved and straight probes

Endocyclophotocoagulation is carried out using a probe attached to a laser unit (Endo Optiks, Little Silver, NJ) which incorporates a diode laser [Figure 1]. Pulsed continuous wave energy is emitted at 810 nm, using a 175 W variable xenon light source, a helium-neon laser aiming beam and video camera imaging. All elements are transmitted via fiberoptics within the probe [Figure 2]. The 20 gauge probe provides a 70° field of view; the 18 gauge probe provides a 110° field. Power, duration, aiming beam intensity, and illumination are adjustable using controls on the console [Figure 1]. A foot pedal controls laser firing; the duration of treatment depends on how long the pedal is depressed.

## TECHNIQUE

**Figure 3:**
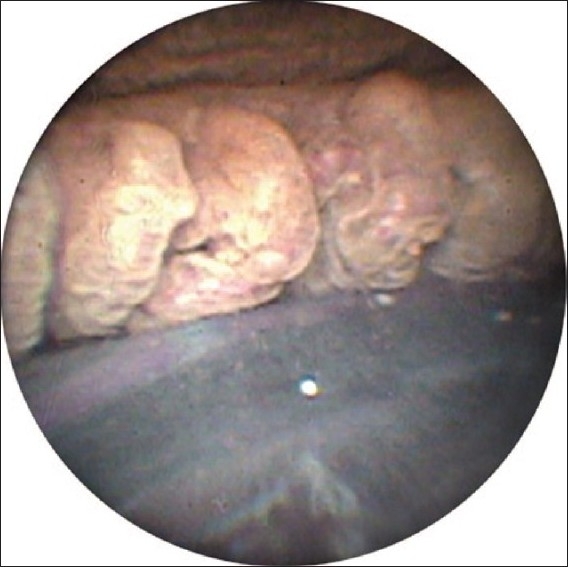
View of ciliary processes

**Figure 4:**
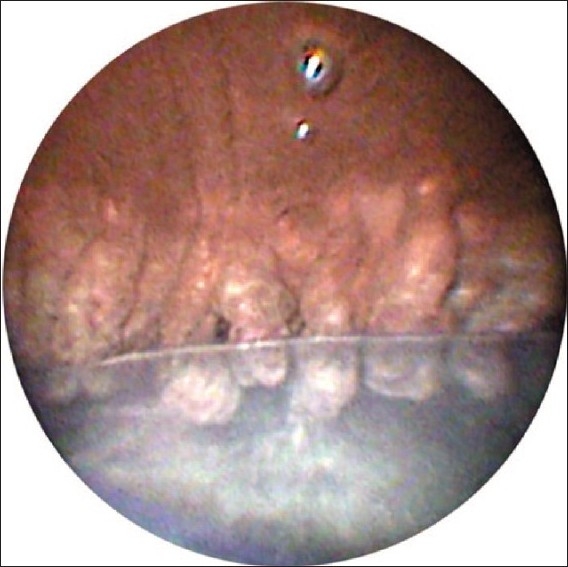
View of ciliary processes over posterior chamber intraocular lens

A retrobulbar or subtenons block with lidocaine and bupivicaine is preferred, but topical anesthesia using intracameral lidocaine is acceptable. The pupil is dilated and a viscoelastic agent is used to expand the ciliary sulcus. After orientation of the probe image outside the eye, the probe is inserted intraocularly through a limbal incision. In select cases, such as posterior synechiae or corneal disease, a pars plana wound can be used in combination with an anterior vitrectomy. After visualization of the ciliary processes using the video monitor [Figures 3 and 4], treatment begins. The laser is set on continuous wave and the energy setting is titrated, usually beginning at 250 mW. Laser energy is applied to each process until shrinkage and whitening occur. The optimal distance between the laser probe and tissue is thought to be about 2 mm.^10^ Following treatment, viscoelastic is removed using irrigation and aspiration followed by wound closure.

Typically, 200-260 degrees can be effectively treated through one incision. A recent study compared one incision versus two incisions in order to effectively treat 360 degrees of the ciliary processes. Better long-term IOP control and less dependence on topical glaucoma medications was noted in the two incision group.^8^ In cases combined with cataract surgery, the probe is inserted in the clear corneal wound and the treatment is performed after placement of the intraocular lens, prior to viscoelastic removal.

## EFFICACY

The largest published series on ECP to date reported retrospectively on cases of refractory glaucoma.^11^ Sixty-eight eyes with glaucoma were treated with ECP; the mean pressure reduction was about 10 mm Hg and the medication usage decreased from 3 to 2. The best-corrected visual acuity was stable or improved in 94% of patients while 6% lost 2 or more lines of vision. The most common complications, included fibrin exudation (24%) and hyphema (12%). Cystoid macular edema was noted in 10%, choroidal detachment in 4%, and there was one case of malignant glaucoma. No cases of phthisis, hypotony, endophthalmitis, retinal detachment or sympathetic ophthalmia were reported.

Uram reported a small prospective series of ten patients who underwent combined phacoemulsification and intraocular lens implantation with endoscopic ciliary process photocoagulation.^12^ All ten patients had uncontrolled open angle glaucoma and cataract. Following phacoemulsification, 180 degrees of ciliary processes were treated before insertion of the posterior chamber intraocular lens. IOP decreased from 31.4 mmHg preoperatively to 13.5 mmHg postoperatively at 19 months follow up.

In a small randomized prospective study of 58 patients who underwent combined cataract and glaucoma surgery, Gayton *et al*. compared the efficacy and safety profiles of trabeculectomy and ECP.^13^ Success was defined as IOP < 19 mmHg without medication. In the trabeculectomy group 42% met this goal, 54% were controlled with medication and 4% were not controlled. In the ECP group 30% met the goal, 65% were controlled with medication and 4% were not controlled. The ECP group had much quieter eyes than those in the trabeculectomy group. The authors concluded that ECP performed through a cataract incision was a reasonably safe and effective alternative to combined cataract and trabeculectomy surgery in patients with glaucoma requiring surgical intervention.

Lima *et al*. performed a prospective comparative study between ECP and Ahmed tube shunt implants in refractory glaucoma.^14^ The success rate was equivalent with both modalities. Sixty-eight pseudophakic eyes with a history of at least one trabeculectomy with an antimetabolite, an IOP of at least 35 mmHg on maximum tolerated medical therapy and visual acuity at least light perception were included. Eyes with a previous history of glaucoma drainage devices or cycloablative procedures were excluded. The mean preoperative IOP was similar in both groups. Average follow-up was 21 months for the ECP group and 19 months for the Ahmed group. With success defined as IOP between 6 and 21 mmHg with or without topical glaucoma therapy, over 70% of patients in each group were successful, no statistical significance between the groups. There were more complications and worsening of visual acuity in the Ahmed group.

Berke and colleagues reviewed 808 patients who underwent phacoemulsification alone and phacoemulsification combined with ECP.^6^,^7^ They showed that combined ECP and phacoemulsification was effective in decreasing the long term need for glaucoma medications in the setting of medically controlled glaucoma at the time of cataract surgery while phacoemulsification alone was not. There was no increased risk of complications. Cystoid macular edema occurred in about 1% of patients in both groups.

Another large prospective study followed 5,824 g laucoma patients for a mean of 5.2 years after they were treated ECP and showed low rates of complications.^15^

Few eyes developed serious long-term complications such as choroidal hemorrhage (n = 5; 0.09%), hypotony/phthisis (n = 7; 0.12%), or progression to no light perception (n = 7; 0.12%). These problems were associated only with neovascular glaucoma or intraoperative hypotony in single-chamber eyes with refractive glaucoma. The rates of other complications were also low; postoperative IOP spikes - 14.5%, hyphema or vitreous hemorrhage - 3.8%, and serous choroidal effusion - 0.36%, regardless of the mechanism of glaucoma. ECP, a cyclodestructive procedure, appeared to be associated with a high rate of development of cataracts - 24.5% (261 of 1,066) of eyes.

ECP has been used extensively in the pediatric population. There have been several studies published regarding the efficacy of ECP as a treatment for pediatric and aphakic glaucoma.^16^–^19^

IOP lowering typically occurs between 1 and 4 weeks after treatment,^11^,^13^,^14^ although exceptions have been noted.^20^

## CONCLUSIONS

Histopathologic studies have confirmed that ECP causes less tissue disruption than transcleral cyclophotocoagulation while still destroying the ciliary body epithelium.^3^–^5^ Although there are no clinical trials favoring ECP over transcleral cyclophotocoagulation, direct visualization of the ciliary processes allows a targeted, titratable approach with fewer associated side effects.^11^–^15^,^21^

ECP is an effective procedure for IOP-lowering in a variety of glaucomas. For most practitioners, the most likely use of ECP is as an adjunct to cataract surgery in the face of moderately uncontrolled glaucoma, but it remains controversial as a primary procedure.

## References

[1] Allingham RR, Damji K, Freedman S, Moroi S, Shafranov G (2005). Chapter 43. Cyclodestructive Surgery. Shields Textbook of Glaucoma.

[2] Kahook MY, Noecker RJ, Schuman JS (2008). Cyclophotocoagulation. Albert and Jakobiec's Principles and Practice of Ophthalmology.

[3] Pantcheva MB, Kahook MK, Schuman JS, Noecker RJ (2007). Comparison of acute structural and histopathological changes in human autopsy eyes after endoscopic cyclophotocoagulation and trans-scleral cyclophotocoagulation. Br J Ophthalmol.

[4] Pantcheva MB, Kahook MY, Schuman JS, Rubin MW, Noecker RJ (2007). Comparison of acute structural and histopathological changes of the porcine ciliary processes after endoscopic cyclophotocoagulation and transcleral cyclophotocoagulation. Clin Experiment Ophthalmol.

[5] Lin SC, Chen MJ, Lin MS, Howes E, Stamper RL (2006). Vascular effects of ciliary tissue from endoscopic versus trans-scleral cyclophotocoagulation. Br J Opthalmol.

[6] Berke SJ, Sturm RT, Caronia RM, Nelson DB, D'Aversa G, Freedman M (2006). Phacoemulsification combined with endoscopic cyclophotocoagulation (ECP) in the management of cataract and medically controlled glaucoma: A large, long term study American Glaucoma Society 16^th^ Annual Meeting.

[7] Berke SJ, Sturm RT, Caronia RM, Nelson DB (2006). Phacoemulsification combined with endoscopic cyclophotocoagulation in the management of cataract and glaucoma. American Academy of Ophthalmology, Annual Meeting, November 2006.

[8] Kahook MY, Lathrop KL, Noecker RJ (2007). One-site versus two-site endoscopic cyclophotocoagulation. J Glaucoma.

[9] Kahook MY, Schuman JS, Noecker RJ (2007). Endoscopic cyclophotocoagulation using iris hooks versus viscoelastic devices. Ophthalmic Surg Lasers Imaging.

[10] Yu JY, Kahook MY, Lathrop KL, Noecker RJ (2008). The effect of probe placement and type of viscoelastic material on endoscopic cyclophotocoagulation laser energy transmission. Ophthalmic Surg Lasers Imaging.

[11] Chen J, Cohn RA, Lin SC, Cortes AE, Alvarado JA (1997). Endoscopic photocoagulation of the ciliary body for treatment of refractory glaucomas. Am J Ophthalmol.

[12] Uram M (1995). Combined phacoemulsification, endoscopic ciliary process photocoagulation, and intraocular lens implantaion in glaucoma management. Ophthalmic Surg.

[13] Gayton JL, Van De Karr M, Sanders V (1999). Combined cataract and glaucoma surgery: Trabeculectomy vs endoscopic laser cycloablation. J Cat Refractive Surg.

[14] Lima FE, Magacho L, Carvalho DM, Susanna R, Avila MP (2004). A prospective, comparative study between endoscopic cyclophotocoagulation and the Ahmed drainage implant in refractory glaucoma. J Glaucoma.

[15] Noecker RJ (2007). Complications of endoscopic cyclophotocoagulation: ECP Collaborative Study Group. Paper presented at: The ASCRS Symposium on Cataract, IOL and Refractive Surgery.

[16] Carter BC, Plager DA, Neely DE, Sprunger DT, Sondhi N, Roberts GJ (2007). Endoscopic diode laser cyclophotocoagulation in the management of aphakic and pseudophakic glaucoma in children. J AAPOS.

[17] Al-Haddad CE, Freedman SF (2007). Endoscopic laser cyclophotocoagulation in pediatric glaucoma with corneal opacities. J AAPOS.

[18] Neely DE, Plager DA (2001). Endocyclophotocoagulation for the management of difficult pediatric glaucomas. J AAPOS.

[19] Barkana Y, Morad Y, Ben-nun J (2002). Endoscopic photocoagulation of the ciliary body after repeated failure of trans-scleral diode-laser photocoagulation. Am J Ophthalmol.

[20] Hollander DA, Lin SC (2003). Delayed therapeutic success with endoscopic cyclophotocoagulation in treating refractory post-penetrating keratoplasty glaucoma. Br J Ophthalmol.

[21] Bloom PA, Dharmaraj S (2006). Endoscopic and transcleral cyclophotocoagulation. Br J Ophthalmol.

